# Supplementary material for: Leucine inhibits degradation of outer mitochondrial membrane proteins to adapt mitochondrial respiration
Source: Nat Cell Biol. 2025 Oct 31;27(11):1889–901. doi: 10.1038/s41556-025-01799-3 (PMC12611767; doi:10.1038/s41556-025-01799-3)
Supplement: Supplementary file 1 — Supplementary Tables 1–4. [file 41556_2025_1799_MOESM1_ESM.pdf]

nature cell biology

Supplementary information https://doi.org/10.1038/s41556-025-01799-3

Leucine inhibits degradation of outer mitochondrial membrane proteins to adapt mitochondrial respiration

In the format provided by the authors and unedited

Supplementary Table 1. Mitochondrial genes tested in Extended Data Fig. 2c.

MitoPathway		Gene Name		
Mitochondrial central dogma	mtDNA maintenance	*polg-1*		
	mtRNA metabolism	*mma-1*	*hoe-1*	
	Translation	*gfm-1*	*mrps-5*	*mrrf-1*
Protein import, sorting and homeostasis	TOM	*tomm-20*	*tomm-22*	*tomm-40*
	MIA40	*wah-1*		
	import motor	*dnj-21*		
	OXA	*oxa-1*		
	Translocase	*mspn-1*		
	Proteases	*clpp-1*	*lonp-1*	
	Chaperones	*fkb-3*		
OXPHOS	Complex I	*nuo-1*	*gas-1*	
	Complex II	*mev-1*	*sdhb-1*	
	Complex III	*cyc-1*	*isp-1*	
	Complex IV	*cco-1*	*cox-4*	
	Complex V	*atp-3*		
Small molecule transport	SCL25A family	*slc-25A26*	*slc-25A42*	*slc-25A46*
Signalling	Calcium homeostasis	*mcu-1*	*vdac-1*	*ncx-6*
	Immune response	*cri-3*		
	cAMP-PKA signalling	*rgs-5*		
Mitochondrial dynamics and surveillance	Fusion	*fzo-1*	*eat-3*	
	Fission	*drp-1*		
	Intramitochondrial membrane interactions	*mtx-1*		
	Trafficking	*hum-2*		

Supplementary Table 2. List of genes encoding amino acid metabolic enzymes tested in Fig. 2a.

WormBase ID	Common Name
WBGene00016652	*got-2.1*
WBGene00020382	*T09B4.8*
WBGene00020347	*ech-1.2*
WBGene00008127	*ddo-1*
WBGene00017301	*F09F7.4*
WBGene00012909	*spds-1*
WBGene00007509	*C10C5.5*
WBGene00010794	*dld-1*
WBGene00001149	*bcat-1*
WBGene00009636	*F42F12.4*
WBGene00017084	*E01A2.1*
WBGene00010383	*mpst-3*
WBGene00009048	*cth-1*
WBGene00018357	*moc-3*
WBGene00009319	*mccc-1*
WBGene00021128	*W10C8.5*
WBGene00010035	*F54C8.1*
WBGene00013379	*Y62E10A.13*
WBGene00001603	*gln-2*
WBGene00012713	*Y39E4A.3*
WBGene00020679	*ogdh-1*
WBGene00010380	*mpst-2*
WBGene00000239	*bas-1*
WBGene00020842	*T27A3.6*
WBGene00020812	*acdh-7*
WBGene00003234	*mif-1*
WBGene00001794	*gta-1*
WBGene00020149	*T01D1.4*
WBGene00000112	*alh-6*
WBGene00000117	*alh-11*
WBGene00001155	*ech-6*
WBGene00009271	*glna-3*
WBGene00015332	*tyr-1*
WBGene00000800	*cars-1*
WBGene00016333	*C32F10.8*
WBGene00003162	*mdh-2*
WBGene00000181	*ard-1*
WBGene00001790	*gst-42*
WBGene00019962	*cysl-3*
WBGene00019819	*R02D3.1*

WBGene00011932	*sptl-3*
WBGene00022856	*cth-2*
WBGene00018398	*sptl-2*
WBGene00016509	*adss-1*
WBGene00020436	*T12A2.1*
WBGene00011291	*R102.4*
WBGene00013870	*ZC373.5*
WBGene00007197	*B0513.5*
WBGene00011064	*adsl-1*
WBGene00008409	*mpst-1*
WBGene00010924	*M153.1*
WBGene00006416	*sams-5*
WBGene00018491	*mdh-1*
WBGene00001158	*ech-9*
WBGene00020950	*dlst-1*
WBGene00004025	*phy-2*
WBGene00019096	*cysl-4*
WBGene00008732	*F13B12.4*
WBGene00000108	*alh-2*
WBGene00017765	*gcst-1*
WBGene00017648	*ddo-3*
WBGene00011587	*T07F10.1*
WBGene00007756	*C27A7.5*
WBGene00012855	*Y44A6D.5*
WBGene00022104	*acsd-1*
WBGene00004259	*pyr-1*
WBGene00003816	*asns-1*
WBGene00000107	*alh-1*
WBGene00009918	*F52A8.5*
WBGene00001077	*dpy-18*
WBGene00011089	*kmo-1*
WBGene00016943	*acdh-1*
WBGene00020366	*acdh-10*
WBGene00001602	*gln-1*
WBGene00015326	*ivd-1*
WBGene00008354	*gcsh-1*
WBGene00008205	*sams-1*
WBGene00002250	*lap-2*
WBGene00008435	*glna-2*
WBGene00021787	*Y51H7C.9*
WBGene00007507	*C10C5.3*
WBGene00007533	*cbl-1*

WBGene00019433	*acdh-3*
WBGene00015334	*sup-18*
WBGene00015538	*sams-3*
WBGene00011938	*alh-13*
WBGene00010286	*F58H1.3*
WBGene00006562	*tdc-1*
WBGene00000138	*amx-2*
WBGene00004026	*phy-3*
WBGene00011307	*mpst-7*
WBGene00015540	*sams-4*
WBGene00007236	*C01G10.9*
WBGene00001157	*ech-8*
WBGene00000114	*alh-8*
WBGene00020146	*got-1.2*
WBGene00008514	*F02A9.4*
WBGene00010456	*K01C8.1*
WBGene00020797	*T25D3.3*
WBGene00007653	*cysl-1*
WBGene00011767	*agxt-1*
WBGene00016294	*C31H2.4*
WBGene00010941	*gss-1*
WBGene00000118	*alh-12*
WBGene00017387	*mpst-4*
WBGene00000111	*alh-5*
WBGene00018764	*F53F10.2*
WBGene00017442	*F13H8.9*
WBGene00015778	*got-2.2*
WBGene00013220	*ctl-3*
WBGene00010661	*tyr-2*
WBGene00007508	*C10C5.4*
WBGene00001150	*ech-1.1*
WBGene00000113	*alh-7*
WBGene00018783	*cbs-2*
WBGene00016020	*sptl-1*
WBGene00006541	*tbh-1*
WBGene00000296	*cat-2*
WBGene00016629	*C44B7.7*
WBGene00000831	*ctl-2*
WBGene00009001	*tyr-3*
WBGene00016419	*tyr-4*
WBGene00001527	*gcs-1*
WBGene00002249	*lap-1*

WBGene00000115	*alh-9*
WBGene00006600	*tph-1*
WBGene00015733	*C13B9.2*
WBGene00009232	*nkat-1*
WBGene00001843	*hgo-1*
WBGene00007129	*B0272.3*
WBGene00021553	*tyr-5*
WBGene00003214	*mel-32*
WBGene00015814	*C16A3.10*
WBGene00019406	*acdh-8*
WBGene00016201	*tdo-2*

Supplementary Table 3.

**Full list of GO terms enriched in Leu_CHX vs. CHX in *C. elegans***				
**GO term**	**Description**	P-value	FDR q-value	Enrichment (N, B, n, b)
GO:0006839	mitochondrial transport	5.92E-09	2.53E-05	3.69 (2937,27,619,21)
GO:0055085	transmembrane transport	5.21E-08	1.11E-04	2.31 (2937,191,340,51)
GO:1990542	mitochondrial transmembrane transport	7.67E-08	1.09E-04	3.61 (2937,25,619,19)
GO:0006811	ion transport	2.36E-06	2.53E-03	2.61 (2937,137,271,33)
GO:0015867	ATP transport	9.84E-06	8.42E-03	7.58 (2937,8,339,7)
GO:0015893	drug transport	1.31E-05	9.35E-03	4.05 (2937,13,613,11)
GO:0015711	organic anion transport	3.46E-05	2.11E-02	3.25 (2937,48,339,18)
GO:0006855	drug transmembrane transport	3.65E-05	1.95E-02	5.11 (2937,9,511,8)
GO:0006862	nucleotide transport	4.55E-05	2.16E-02	6.74 (2937,9,339,7)
GO:0051503	adenine nucleotide transport	4.55E-05	1.95E-02	6.74 (2937,9,339,7)
GO:0015868	purine ribonucleotide transport	4.55E-05	1.77E-02	6.74 (2937,9,339,7)
GO:0015865	purine nucleotide transport	4.55E-05	1.62E-02	6.74 (2937,9,339,7)
GO:0006820	anion transport	6.94E-05	2.28E-02	3.20 (2937,61,271,18)
GO:0051179	localization	1.22E-04	3.74E-02	1.23 (2937,588,987,243)
GO:0015748	organophosphate ester transport	1.52E-04	4.34E-02	4.87 (2937,16,339,9)
GO:0006810	transport	2.31E-04	6.19E-02	1.57 (2937,496,271,72)
GO:0051234	establishment of localization	2.75E-04	6.91E-02	1.23 (2937,528,987,219)
GO:1990544	mitochondrial ATP transmembrane transport	4.87E-04	1.16E-01	8.66 (2937,4,339,4)
GO:0015866	ADP transport	4.87E-04	1.10E-01	8.66 (2937,4,339,4)
GO:0140021	mitochondrial ADP transmembrane transport	4.87E-04	1.04E-01	8.66 (2937,4,339,4)
GO:0006865	amino acid transport	8.13E-04	1.66E-01	5.61 (2937,16,229,7)
GO:0007005	mitochondrion organization	8.78E-04	1.71E-01	1.85 (2937,45,989,28)
GO:0000041	transition metal ion transport	9.63E-04	1.79E-01	104.89 (2937,14,4,2)
**Full list of GO terms enriched in Leu_CHX vs. CHX in HEK293 cells**				
**GO term**	**Description**	P-value	FDR q-value	Enrichment (N, B, n, b)
GO:0006082	organic acid metabolic process	2.15E-07	1.68E-04	1.53 (4737,428,994,137)
GO:0006091	generation of precursor metabolites and energy	3.40E-08	3.39E-05	1.88 (4737,188,993,74)

GO:0006099	tricarboxylic acid cycle	3.32E-05	1.35E-02	3.02 (4737,29,919,17)
GO:0006101	citrate metabolic process	6.15E-05	2.18E-02	2.92 (4737,30,919,17)
GO:0006102	isocitrate metabolic process	9.79E-04	1.63E-01	5.15 (4737,5,919,5)
GO:0006120	mitochondrial electron transport, NADH to ubiquinone	2.92E-04	6.97E-02	3.57 (4737,39,442,13)
GO:0006122	mitochondrial electron transport, ubiquinol to cytochrome c	2.19E-04	5.58E-02	5.29 (4737,9,696,7)
GO:0006213	pyrimidine nucleoside metabolic process	2.21E-04	5.50E-02	4.84 (4737,19,464,9)
GO:0006353	DNA-templated transcription, termination	3.28E-04	7.33E-02	43.86 (4737,27,12,3)
GO:0006414	translational elongation	1.31E-09	1.80E-06	2.72 (4737,101,741,43)
GO:0006415	translational termination	1.82E-11	5.00E-08	3.02 (4737,91,741,43)
GO:0006520	cellular amino acid metabolic process	8.45E-04	1.45E-01	1.63 (4737,161,994,55)
GO:0006839	mitochondrial transport	1.79E-04	4.90E-02	2.07 (4737,104,748,34)
GO:0007175	negative regulation of epidermal growth factor-activated receptor activity	2.49E-04	6.07E-02	26.12 (4737,4,136,3)
GO:0008152	metabolic process	4.30E-11	9.43E-08	1.16 (4737,2828,994,689)
GO:0008284	positive regulation of cell proliferation	7.79E-04	1.42E-01	6.08 (4737,188,29,7)
GO:0008535	respiratory chain complex IV assembly	5.35E-05	1.96E-02	4.51 (4737,22,525,11)
GO:0009058	biosynthetic process	3.09E-07	2.26E-04	1.32 (4737,969,967,262)
GO:0009060	aerobic respiration	1.12E-06	6.45E-04	4.00 (4737,33,610,17)
GO:0009063	cellular amino acid catabolic process	7.35E-04	1.37E-01	2.13 (4737,60,928,25)
GO:0010257	NADH dehydrogenase complex assembly	2.10E-06	1.00E-03	3.62 (4737,57,459,20)
GO:0015980	energy derivation by oxidation of organic compounds	1.37E-04	3.95E-02	2.40 (4737,57,832,24)
GO:0016999	antibiotic metabolic process	4.81E-05	1.82E-02	2.29 (4737,60,964,28)
GO:0017004	cytochrome complex assembly	8.96E-05	2.98E-02	3.53 (4737,31,606,14)
GO:0017144	drug metabolic process	3.26E-04	7.44E-02	1.52 (4737,239,991,76)
GO:0018126	protein hydroxylation	8.03E-05	2.75E-02	3.28 (4737,19,988,13)
GO:0019511	peptidyl-proline hydroxylation	1.11E-04	3.30E-02	5.15 (4737,11,669,8)
GO:0019752	carboxylic acid metabolic process	5.55E-06	2.44E-03	1.49 (4737,390,994,122)

GO:0022411	cellular component disassembly	1.81E-04	4.84E-02	1.58 (4737,207,982,68)
GO:0022900	electron transport chain	4.88E-08	4.46E-05	2.23 (4737,105,993,49)
GO:0022904	respiratory electron transport chain	1.68E-09	2.05E-06	3.46 (4737,76,558,31)
GO:0031134	sister chromatid biorientation	8.44E-04	1.49E-01	1,184.25 (4737,1,4,1)
GO:0032964	collagen biosynthetic process	3.63E-04	7.95E-02	17.42 (4737,3,272,3)
GO:0032981	mitochondrial respiratory chain complex I assembly	2.10E-06	1.05E-03	3.62 (4737,57,459,20)
GO:0032984	protein-containing complex disassembly	3.10E-06	1.42E-03	2.05 (4737,156,741,50)
GO:0033108	mitochondrial respiratory chain complex assembly	7.66E-12	2.80E-08	3.69 (4737,88,525,36)
GO:0033617	mitochondrial respiratory chain complex IV assembly	5.75E-04	1.17E-01	4.27 (4737,19,525,9)
GO:0043392	negative regulation of DNA binding	7.35E-04	1.39E-01	26.76 (4737,9,59,3)
GO:0043436	oxoacid metabolic process	4.57E-07	3.13E-04	1.51 (4737,425,994,135)
GO:0043624	cellular protein complex disassembly	8.06E-08	6.80E-05	2.43 (4737,113,741,43)
GO:0043933	protein-containing complex subunit organization	3.52E-05	1.38E-02	1.41 (4737,742,648,143)
GO:0044237	cellular metabolic process	1.18E-10	1.85E-07	1.17 (4737,2713,994,664)
GO:0044249	cellular biosynthetic process	7.17E-07	4.37E-04	1.32 (4737,903,994,251)
GO:0044271	cellular nitrogen compound biosynthetic process	9.23E-05	2.98E-02	1.35 (4737,564,967,156)
GO:0044281	small molecule metabolic process	7.17E-11	1.31E-07	1.47 (4737,742,994,229)
GO:0044283	small molecule biosynthetic process	1.05E-04	3.19E-02	1.56 (4737,236,991,77)
GO:0045333	cellular respiration	6.75E-07	4.35E-04	3.78 (4737,39,610,19)
GO:0045893	positive regulation of transcription, DNA-templated	3.76E-04	7.93E-02	5.66 (4737,291,23,8)
GO:0046487	glyoxylate metabolic process	6.42E-04	1.26E-01	5.61 (4737,5,844,5)
GO:0051186	cofactor metabolic process	9.99E-05	3.13E-02	1.57 (4737,248,938,77)
GO:0051188	cofactor biosynthetic process	6.48E-04	1.25E-01	1.78 (4737,119,938,42)
GO:0055114	oxidation-reduction process	6.74E-09	7.39E-06	1.86 (4737,216,993,84)
GO:0070125	mitochondrial translational elongation	2.90E-12	3.18E-08	3.20 (4737,84,741,42)
GO:0070126	mitochondrial translational termination	4.88E-12	2.67E-08	3.16 (4737,85,741,42)
GO:0070816	phosphorylation of RNA polymerase II C-terminal domain	8.29E-04	1.49E-01	2,368.50 (4737,2,1,1)

GO:0071704	organic substance metabolic process	1.66E-06	8.66E-04	1.13 (4737,2642,992,627)
GO:0072350	tricarboxylic acid metabolic process	1.88E-04	4.91E-02	2.74 (4737,32,919,17)
GO:1901564	organonitrogen compound metabolic process	1.44E-04	4.05E-02	1.16 (4737,1779,991,432)
GO:1901566	organonitrogen compound biosynthetic process	3.27E-05	1.38E-02	1.41 (4737,479,959,137)
GO:1901576	organic substance biosynthetic process	1.24E-06	6.78E-04	1.31 (4737,951,994,261)
GO:1901661	quinone metabolic process	6.16E-04	1.23E-01	3.18 (4737,17,964,11)
GO:1902680	positive regulation of RNA biosynthetic process	3.94E-04	8.15E-02	5.64 (4737,292,23,8)
GO:1903508	positive regulation of nucleic acid-templated transcription	3.76E-04	8.09E-02	5.66 (4737,291,23,8)
GO:1904742	regulation of telomeric DNA binding	8.59E-04	1.45E-01	53.53 (4737,3,59,2)
GO:1904743	negative regulation of telomeric DNA binding	3.05E-04	7.12E-02	80.29 (4737,2,59,2)
GO:1990758	mitotic sister chromatid biorientation	8.44E-04	1.47E-01	1,184.25 (4737,1,4,1)

Supplementary Table 4. *C. elegans* strains used in this study.

Strain name	Source	Identifier
C. elegans: N2 wild type	CGC	WormBase ID: WBStrain00000001
C. elegans: PP4027: *unc-119(ed4); hhIs286[Peft-3::UbV::GFP::fis-1-TM::unc-54 3'UTR; unc-119+] II*	This study	N/A
C. elegans: SJZ328: *foxSi75 [eft-3p::tomm-20::mKate2::HA::tbb-2 3' UTR] I*	CGC	WormBase ID: WBStrain00034085
C. elegans: PP4028: *unc-119(ed4); hhIs286[Peft-3::UbV::GFP::fis-1-TM::unc-54 3'UTR; unc-119+]; foxSi75 [eft-3p::tomm-20::mKate2::HA::tbb-2 3' UTR] I*	This study	N/A
C. elegans: PP4029: *foxSi75 [eft-3p::tomm-20::mKate2::HA::tbb-2 3' UTR] I; unc-119(ed4); hhIs64 [unc-119(+); sur-5::UbiV-GFP] III*	This study	N/A
C. elegans: FX544: *cdc-48.1(tm544) II*	CGC	WormBase ID: WBStrain00007563
C. elegans: PP4031: *cdc-48.1(tm544)II; unc-119(ed4) III; hhIs286[Peft-3::UbV::GFP::fis-1-TM::unc-54 3'UTR; unc-119+] II*	This study	N/A
C. elegans: PP4032: *unc-119(ed4); hhEx203[Peft-3::GFP::fis-1-TM::unc-54 3'UTR; unc-119+]*	This study	N/A
C. elegans: PP4035: *unc-119(ed4); hhIs287[Peft-3::GFP::fis-1-TM::unc-54 3'UTR; unc-119+]*	This study	N/A
C. elegans: PP563: *unc-119(ed4) III; hhIs64[unc-119(+); Psur-5::UbV-GFP] III*	Hoppe lab	N/A
C. elegans: PP4209: *+/szT1[lon-2(e678) umnIs39]I;bcat-1(E279K)/szT1[umnIs40] X*	Hoppe lab	N/A
C. elegans: PP4211: *hhIs286[Peft-3::UbV::GFP::fis-1-TM::unc-54 3'UTR; unc-119+] II; +/szT1[lon-2(e678) umnIs39]I;bcat-1(E279K)/szT1[umnIs40] X*	Hoppe lab	N/A
