# Supplementary material for: Leucine inhibits degradation of outer mitochondrial membrane proteins to adapt mitochondrial respiration
Source: Nat Cell Biol. 2025 Oct 31;27(11):1889–901. doi: 10.1038/s41556-025-01799-3 (PMC12611767; doi:10.1038/s41556-025-01799-3)
Supplement: Supplementary file 2 — Reporting Summary [file 41556_2025_1799_MOESM2_ESM.pdf]

nature portfolio

Corresponding author(s): Prof. Dr. Thorsten Hoppe

Last updated by author(s): Sep 1, 2025

Reporting Summary

Nature Portfolio wishes to improve the reproducibility of the work that we publish. This form provides structure for consistency and transparency in reporting. For further information on Nature Portfolio policies, see our Editorial Policies and the Editorial Policy Checklist.

Statistics

For all statistical analyses, confirm that the following items are present in the figure legend, table legend, main text, or Methods section.

n/a	Confirmed	
☐	☒	The exact sample size (*n*) for each experimental group/condition, given as a discrete number and unit of measurement
☐	☒	A statement on whether measurements were taken from distinct samples or whether the same sample was measured repeatedly
☐	☒	The statistical test(s) used AND whether they are one- or two-sided<br>*Only common tests should be described solely by name; describe more complex techniques in the Methods section.*
☒	☐	A description of all covariates tested
☐	☒	A description of any assumptions or corrections, such as tests of normality and adjustment for multiple comparisons
☐	☒	A full description of the statistical parameters including central tendency (e.g. means) or other basic estimates (e.g. regression coefficient) AND variation (e.g. standard deviation) or associated estimates of uncertainty (e.g. confidence intervals)
☐	☒	For null hypothesis testing, the test statistic (e.g. *F*, *t*, *r*) with confidence intervals, effect sizes, degrees of freedom and *P* value noted<br>*Give P values as exact values whenever suitable.*
☒	☐	For Bayesian analysis, information on the choice of priors and Markov chain Monte Carlo settings
☒	☐	For hierarchical and complex designs, identification of the appropriate level for tests and full reporting of outcomes
☒	☐	Estimates of effect sizes (e.g. Cohen's *d*, Pearson's *r*), indicating how they were calculated

Our web collection on statistics for biologists contains articles on many of the points above.

Software and code

Policy information about availability of computer code

Data collection	Zeiss 2.3 blue edition<br>Image Studio v5.2.5 (LI-COR Biosciences)<br>FlowPilot-ProTM (v1)<br>Digital Micrograph (Gatan)<br>Seahorse Wave 2.6.1
Data analysis	Image Studio v5.2.5 (LI-COR Biosciences)<br>Image Lab v6.1.0 build 7<br>ImageJ v2.1.0/1.53c<br>R3.6.2 and R Studio v1.2.5033<br>Prism v10.1.1<br>Perseus 2.0.10.0<br>Seahorse Wave 2.6.1

For manuscripts utilizing custom algorithms or software that are central to the research but not yet described in published literature, software must be made available to editors and reviewers. We strongly encourage code deposition in a community repository (e.g. GitHub). See the Nature Portfolio guidelines for submitting code & software for further information.

Data

Policy information about availability of data

All manuscripts must include a data availability statement. This statement should provide the following information, where applicable:

- Accession codes, unique identifiers, or web links for publicly available datasets
- A description of any restrictions on data availability
- For clinical datasets or third party data, please ensure that the statement adheres to our policy

Source data of this study are available within the paper, its Supplementary Information, and public repository. The mass spectrometry proteomics data have been deposited to the ProteomeXchange Consortium via the PRIDE partner repository with the dataset identifiers PXD051398, PXD051401, PXD051403, PXD062734, PXD062736. Any other information related to the findings of this manuscript are available from the corresponding author upon request. Plasmids and C. elegans strains generated in this study will be distributed to researchers upon request.

Research involving human participants, their data, or biological material

Policy information about studies with human participants or human data. See also policy information about sex, gender (identity/presentation), and sexual orientation and race, ethnicity and racism.

Reporting on sex and gender	*Use the terms sex (biological attribute) and gender (shaped by social and cultural circumstances) carefully in order to avoid confusing both terms. Indicate if findings apply to only one sex or gender; describe whether sex and gender were considered in study design; whether sex and/or gender was determined based on self-reporting or assigned and methods used. Provide in the source data disaggregated sex and gender data, where this information has been collected, and if consent has been obtained for sharing of individual-level data; provide overall numbers in this Reporting Summary. Please state if this information has not been collected. Report sex- and gender-based analyses where performed, justify reasons for lack of sex- and gender-based analysis.*
Reporting on race, ethnicity, or other socially relevant groupings	*Please specify the socially constructed or socially relevant categorization variable(s) used in your manuscript and explain why they were used. Please note that such variables should not be used as proxies for other socially constructed/relevant variables (for example, race or ethnicity should not be used as a proxy for socioeconomic status). Provide clear definitions of the relevant terms used, how they were provided (by the participants/respondents, the researchers, or third parties), and the method(s) used to classify people into the different categories (e.g. self-report, census or administrative data, social media data, etc.) Please provide details about how you controlled for confounding variables in your analyses.*
Population characteristics	*Describe the covariate-relevant population characteristics of the human research participants (e.g. age, genotypic information, past and current diagnosis and treatment categories). If you filled out the behavioural & social sciences study design questions and have nothing to add here, write "See above."*
Recruitment	*Describe how participants were recruited. Outline any potential self-selection bias or other biases that may be present and how these are likely to impact results.*
Ethics oversight	*Identify the organization(s) that approved the study protocol.*

Note that full information on the approval of the study protocol must also be provided in the manuscript.

Field-specific reporting

Please select the one below that is the best fit for your research. If you are not sure, read the appropriate sections before making your selection.

☒ Life sciences ☐ Behavioural & social sciences ☐ Ecological, evolutionary & environmental sciences

For a reference copy of the document with all sections, see nature.com/documents/nr-reporting-summary-flat.pdf

Life sciences study design

All studies must disclose on these points even when the disclosure is negative.

Sample size	No statistical method was used to predetermine sample sizes. Sample size determination was done according to previous literature and standard C. elegans approaches. Exact sample sizes are stated in the figure legends and at least three biological replicates were performed for each experiment.
Data exclusions	No data was excluded from the analysis.
Replication	All experimental replications were indicated in the figure legends. For proteomic analysis, four biological replicates were performed for each condition each experiment. All other experiments were performed with at least three biological replicates.
Randomization	Randomization was not applicable or relevant in our study. Experiments were performed with worms or cells clearly grouped based on genotypes and treatments.

Blinding	The investigators were not blinded as this is not necessary in our study. All experiments in this study were automated quantified by software with defined settings.

Reporting for specific materials, systems and methods

We require information from authors about some types of materials, experimental systems and methods used in many studies. Here, indicate whether each material, system or method listed is relevant to your study. If you are not sure if a list item applies to your research, read the appropriate section before selecting a response.

Materials & experimental systems

n/a	Involved in the study
☐	☒ Antibodies
☐	☒ Eukaryotic cell lines
☒	☐ Palaeontology and archaeology
☐	☒ Animals and other organisms
☒	☐ Clinical data
☒	☐ Dual use research of concern
☒	☐ Plants

Methods

n/a	Involved in the study
☒	☐ ChIP-seq
☐	☒ Flow cytometry
☒	☐ MRI-based neuroimaging

Antibodies

Antibodies used

Living Colors A.v. GFP Monoclonal Antibody [JL-8] Clontech Cat. # 632380
Monoclonal alpha Tubulin antibody [EP1332Y] Abcam Cat. # ab52866; RRID: AB_869989
VDAC2 Polyclonal antibody Proteintech Cat. # 11663-1-AP
TOMM40 Monoclonal antibody Proteintech Cat. # 66658-1-Ig
Anti-MTCO1 antibody [1D6E1A8] Abcam Cat. # ab14705
Anti-SEL1L (N-terminal) antibody produced in rabbit Sigma-Aldrich Cat. # S3699
SYVN1 (D3O2A) Rabbit mAb Cell Signalling Cat. # 14773
Anti-CDC-48.1 Antibody raised in rabbits Hoppe-lab/Biogenes Berlin Custom antibody
Rabbit ANTI-FLAG polyclonal Sigma-Aldrich Cat. # F 7425
Anti-Ubiquitin Antibody, clone P4D1-A11 Sigma-Aldrich Cat. # 05-944
Anti-SEL-1 Antibody Jarosch-lab, Berlin Custom antibody
IRDye® 680RD Donkey anti-Rabbit IgG Secondary Antibody Li-Cor Cat. # 926-68073; RRID: AB_10954442
IRDye® 800CW Donkey anti-Mouse IgG Secondary Antibody Li-Cor Cat. # 926-32212; RRID: AB_621847
Peroxidase AffiniPure™ Goat Anti-Mouse IgG + IgM (H+L) Jackson ImmunoResearch Laboratories RRID: AB_2338503
Peroxidase AffiniPure™ Mouse Anti-Rabbit IgG (H+L) Jackson ImmunoResearch Laboratories RRID: AB_2339150

Validation

Anti-Ubiquitin Antibody, clone P4D1-A11 Sigma-Aldrich Cat. # 05-944: Routinely evaluated by immunoblot of human recombinant ubiquitin (Catalog # 12-558), and ubiquitinated proteins in acid extracts from HeLa cells. https://www.sigmaaldrich.com/DE/en/product/mm/05944?srsltid=AfmBOoouiDBhelTfQQdOx4Qpe_KNPIpMTro5kRvpPNbq4GA7i3W1Xrlc. Dilution 1:3500
Rabbit ANTI-FLAG polyclonal Sigma-Aldrich Cat. # F 7425: The antibody recognizes the FLAG epitope located on FLAG-tagged fusion proteins at the N-terminus or C-terminus, applying dot blot, immunoblotting, immunoprecipitation and immunocytochemistry assays. https://www.sigmaaldrich.com/DE/en/product/sigma/f7425?srsltid=AfmBOorLqBO_BPwLkbHGhqD-UHHJJa6wYNmmt5vwdPjqCnz7aEB7IDG6. Dilution 1:2500
Anti-CDC-48.1 antibody: Franz et al., Mol.Cell., 2011, PMID: 21981920. Dilution 1:5000
Rabbit monoclonal alpha Tubulin antibody [EP1332Y]: Validated in IHC-P, WB, ICC/IF, Flow Cyt (Intra) and tested in Mouse, Rat, Pig, Human, Drosophila melanogaster samples. https://www.abcam.com/en-kr/products/primary-antibodies/alpha-tubulin-antibody-ep1332y-microtubule-marker-ab52866. Dilution 1:5000
Living Colors A.v. GFP Monoclonal Antibody [JL-8]: Validated by GFP positive and negative worms. Dilution 1:5000
Anti-SEL-1 Antibody Jarosch-lab, Berlin Custom antibody: Validated in previous publications and also with sel-1 mutants by western blot analysis. Dilution 1:8000
VDAC2 Polyclonal antibody Proteintech Cat. # 11663-1-AP: Positive WB detected in mouse brain tissue, human heart tissue, mouse heart tissue, rat brain tissue, rat heart tissue. https://www.ptglab.com/products/VDAC2-Antibody-11663-1-AP.htm?srsltid=AfmBOoq1sDWHKDPtAxDWXXI2o1bFS64QDEbTuqB81IL6ldxh4ZnjnSl5#product-information. Dilution 1:1000
TOMM40 Monoclonal antibody Proteintech Cat. # 66658-1-Ig: Positive WB detected in LNCaP cells, HeLa cells, HEK-293 cells, HepG2 cells, human brain tissue, pig brain tissue, Jurkat cells, HSC-T6 cells, PC-12 cells, NIH/3T3 cells, RAW264.7 cells, K-562 cells, rat brain tissue, mouse brain tissue. https://www.ptglab.com/products/TOMM40-Antibody-66658-1-Ig.htm?srsltid=AfmBOoqEk6yp-usG10-gXtjMXVVWRK6AJ01Xdrs2v3yHw3i8egcp4xWG. Dilution 1:2000
Anti-MTCO1 antibody [1D6E1A8] Abcam Cat. # ab14705: validated for use in Flow Cyt, ICC, IHC-P, WB in human, recombinant fragment samples. https://www.abcam.com/en-us/products/primary-antibodies/mtco1-antibody-1d6e1a8-ab14705?srsltid=AfmBOorQMkhyOAsSZXwdPIWeChiMCTcGuPXMMXflLu2_z9wsf1kovD9x#tab=datasheet. Dilution 1:2000
Anti-SEL1L (N-terminal) antibody produced in rabbit Sigma-Aldrich Cat. # S3699: validated in immunoblotting. immunoprecipitation, immunofluorescence. https://www.sigmaaldrich.com/DE/en/product/sigma/s3699?srsltid=AfmBOooG_AlTRKakLSP3L3ITqDI7_ymXpfdUABkDZaz___PJU6YRZBVZ. Dilution 1:1000
SYVN1 (D3O2A) Rabbit mAb Cell Signalling Cat. # 14773: This antibody has been validated by western blot analysis of extracts from various cell lines. https://www.cellsignal.com/products/primary-antibodies/syvn1-d3o2a-rabbit-mab/14773?srsltid=AfmBOoo5Rm1t7crOVb4m9aAegx1XePxc8NBA_2_XZrSl3HnvcGF-sqe7. Dilution 1:2000.

Eukaryotic cell lines

Policy information about cell lines and Sex and Gender in Research

Cell line source(s)	Human: Flp-In T-REx-293-cell line Invitrogen Cat# R78007<br>Human: HEK293 Sigma-Aldrich Cat# 85120602<br>Human: NCI-H2030 [H2030] ATCC CRL-5914™<br>Human: NCI-H1437 [H1437] ATCC CRL-5872™<br>Human: NCI-H1666 [H1666] ATCC CRL-5885™
Authentication	No additional authentication was performed.
Mycoplasma contamination	The cell line used in this study was confirmed negative for Mycoplasma contamination.
Commonly misidentified lines<br>(See ICLAC register)	No commonly misidentified cell line was used in this study.

Animals and other research organisms

Policy information about studies involving animals; ARRIVE guidelines recommended for reporting animal research, and Sex and Gender in Research

Laboratory animals	Caenorhabditis elegans strains of different genotypes are listed in the Supplementary Table. Animal age is indicated in the Method section.
Wild animals	No wild animals were used in this study.
Reporting on sex	All experiments were performed with hermaphrodite C. elegans.
Field-collected samples	No field-collected samples in this study.
Ethics oversight	No ethical approval or guidance is required.

Note that full information on the approval of the study protocol must also be provided in the manuscript.

Plants

Seed stocks	*Report on the source of all seed stocks or other plant material used. If applicable, state the seed stock centre and catalogue number. If plant specimens were collected from the field, describe the collection location, date and sampling procedures.*
Novel plant genotypes	*Describe the methods by which all novel plant genotypes were produced. This includes those generated by transgenic approaches, gene editing, chemical/radiation-based mutagenesis and hybridization. For transgenic lines, describe the transformation method, the number of independent lines analyzed and the generation upon which experiments were performed. For gene-edited lines, describe the editor used, the endogenous sequence targeted for editing, the targeting guide RNA sequence (if applicable) and how the editor was applied.*
Authentication	*Describe any authentication procedures for each seed stock used or novel genotype generated. Describe any experiments used to assess the effect of a mutation and, where applicable, how potential secondary effects (e.g. second site T-DNA insertions, mosiacism, off-target gene editing) were examined.*

Flow Cytometry

Plots

Confirm that:

☒ The axis labels state the marker and fluorochrome used (e.g. CD4-FITC).

☒ The axis scales are clearly visible. Include numbers along axes only for bottom left plot of group (a 'group' is an analysis of identical markers).

☒ All plots are contour plots with outliers or pseudocolor plots.

☒ A numerical value for number of cells or percentage (with statistics) is provided.

Methodology

Sample preparation	Synchronized day 1 adults by timed-egg laying
Instrument	BioSorter (Union Biometrica)
Software	FlowPilot-ProTM (v1)

Cell population abundance	50-250 gated worms were collected from each independent biological replicate
Gating strategy	Gating of day 1 adults was based on TOF > 1000 and Extinction > 600.

☒ Tick this box to confirm that a figure exemplifying the gating strategy is provided in the Supplementary Information.
