# Supplementary figures and images for: Leucine inhibits degradation of outer mitochondrial membrane proteins to adapt mitochondrial respiration
Source: Nat Cell Biol. 2025 Oct 31;27(11):1889–901. doi: 10.1038/s41556-025-01799-3 (PMC12611767; doi:10.1038/s41556-025-01799-3)

uncropped blots related for Fig. 1:

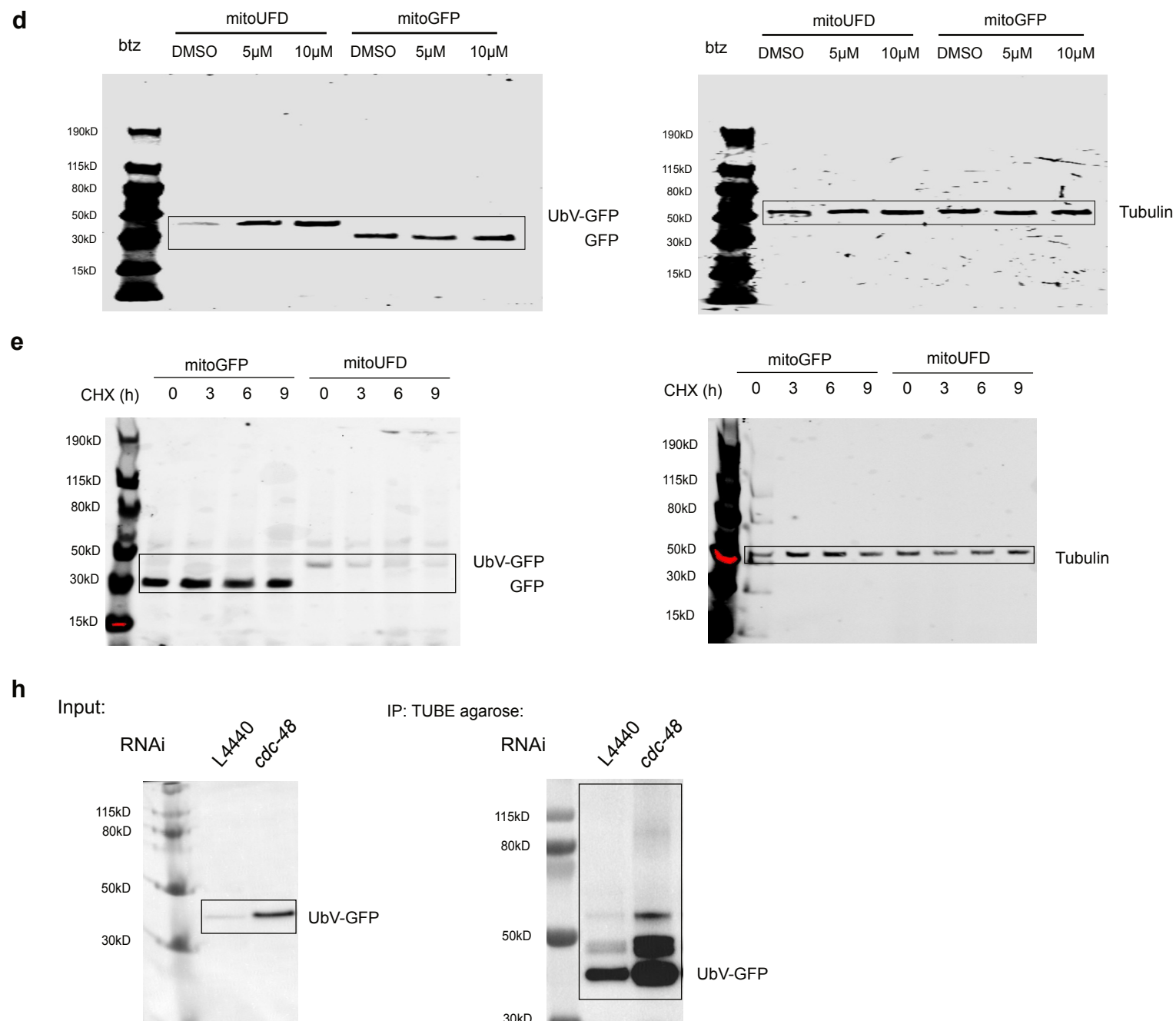

Supplement: Supplementary file 5 — Uncropped blots. [file 41556_2025_1799_MOESM5_ESM.pdf]

uncropped blots related for Fig. 2:

**i**

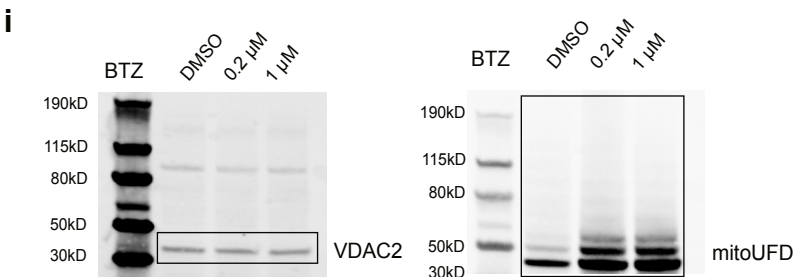

**k**

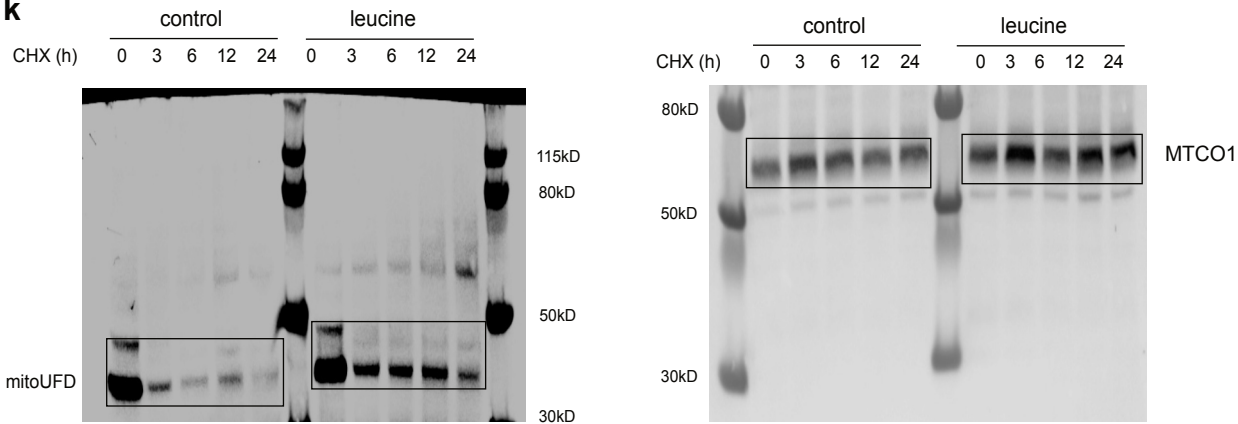

Supplement: Supplementary file 7 — Uncropped blots. [file 41556_2025_1799_MOESM7_ESM.pdf]

uncropped blots related for Fig. 3:

**a**

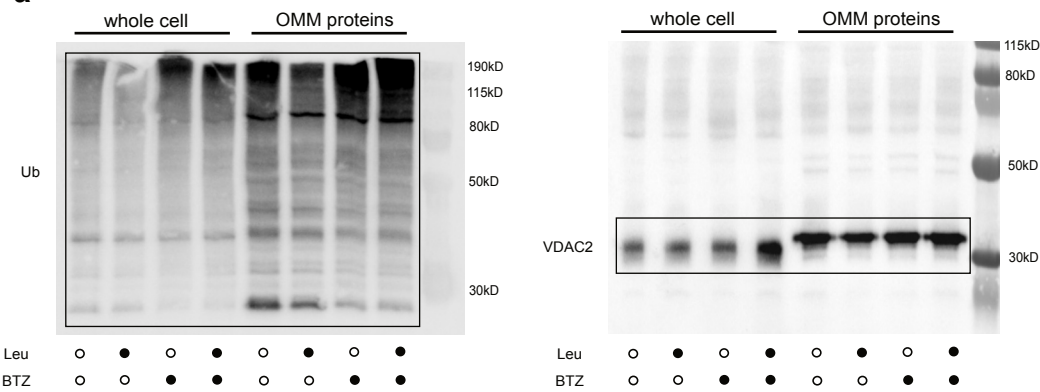

**b**

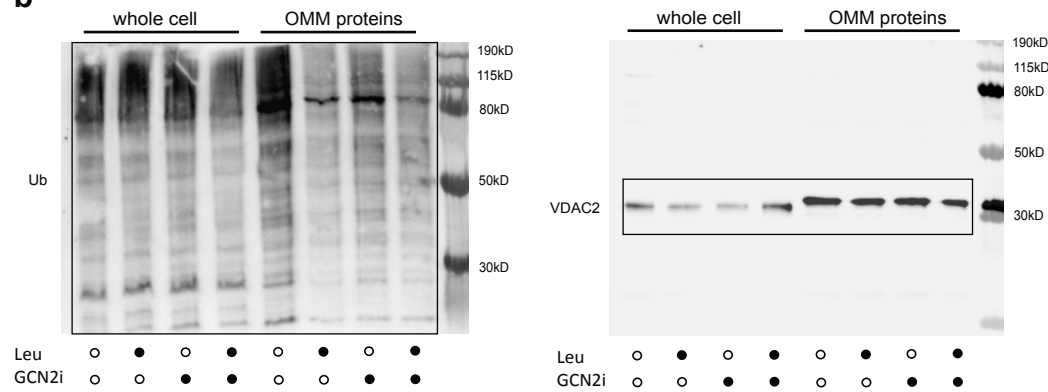

**d**

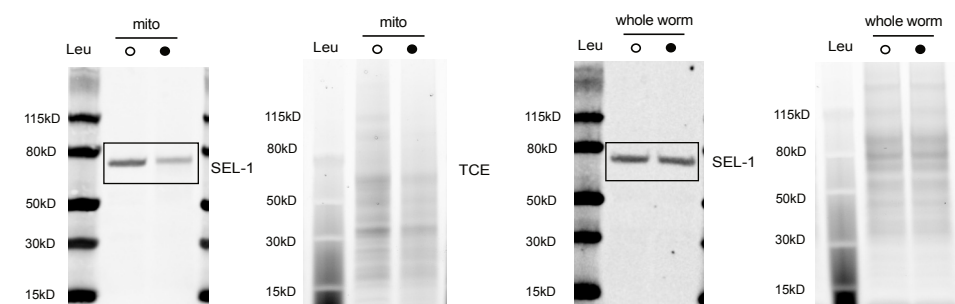

**e**

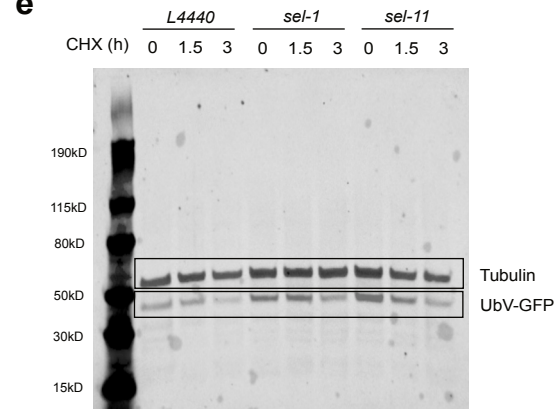

**h**

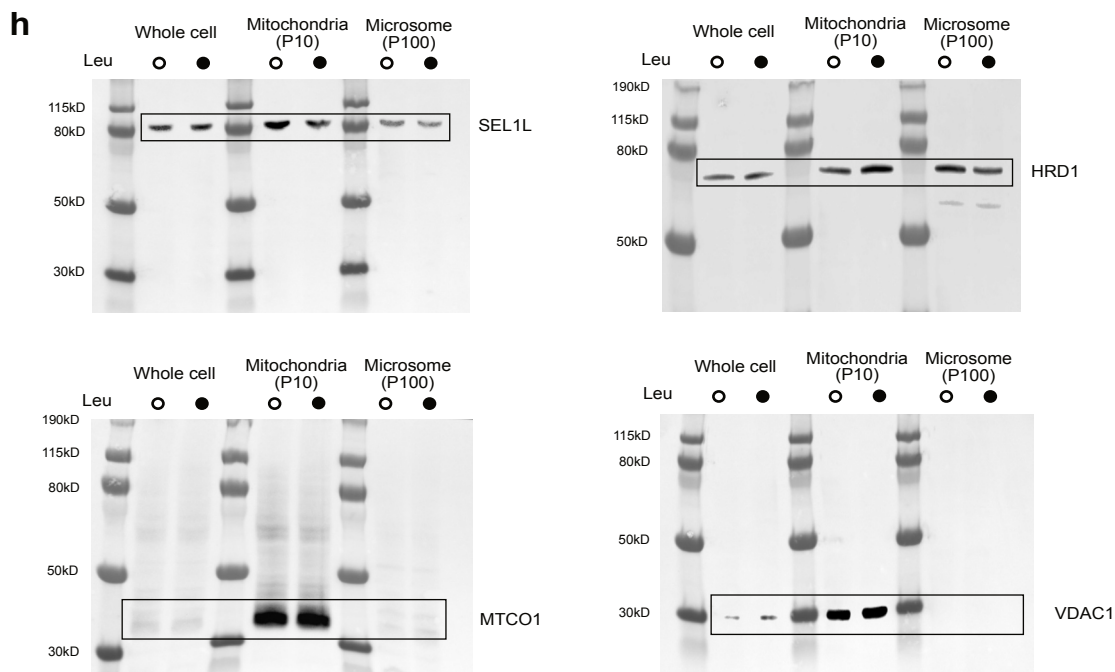

Supplement: Supplementary file 9 — Uncropped blots. [file 41556_2025_1799_MOESM9_ESM.pdf]

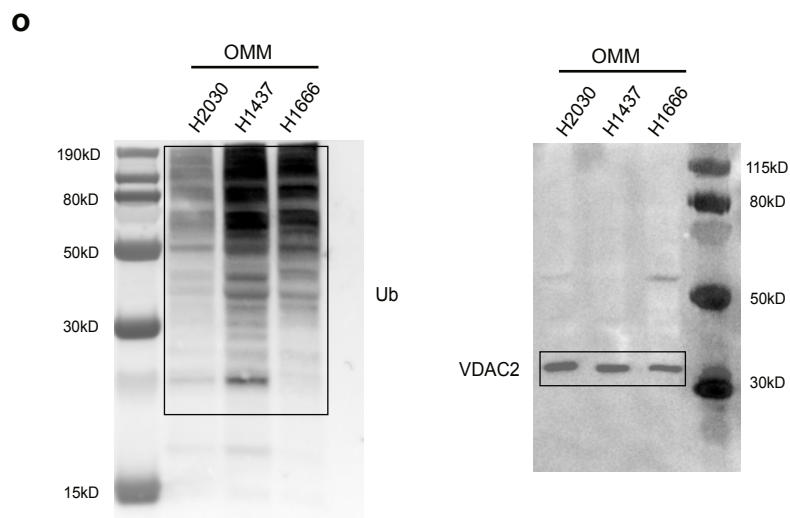

Supplement: Supplementary file 12 — Uncropped blots. [file 41556_2025_1799_MOESM12_ESM.pdf]

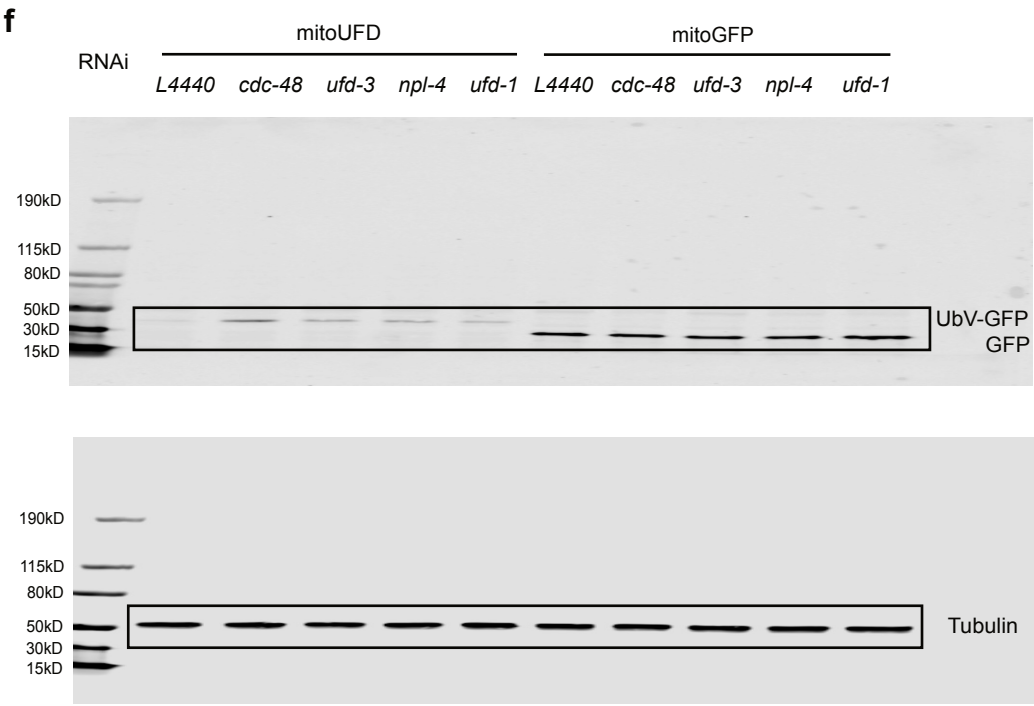

Supplement: Supplementary file 14 — Uncropped blots. [file 41556_2025_1799_MOESM14_ESM.pdf]

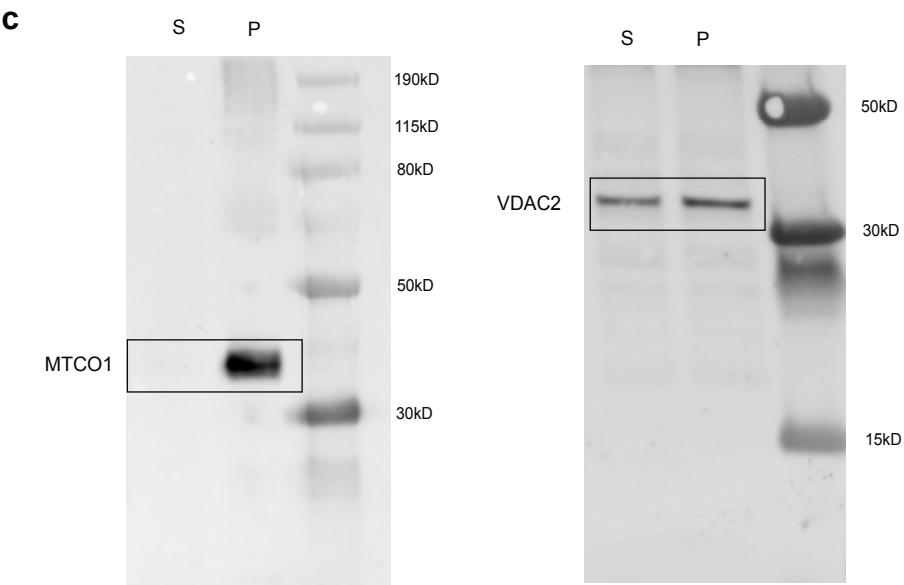

Supplement: Supplementary file 18 — Uncropped blots. [file 41556_2025_1799_MOESM18_ESM.pdf]

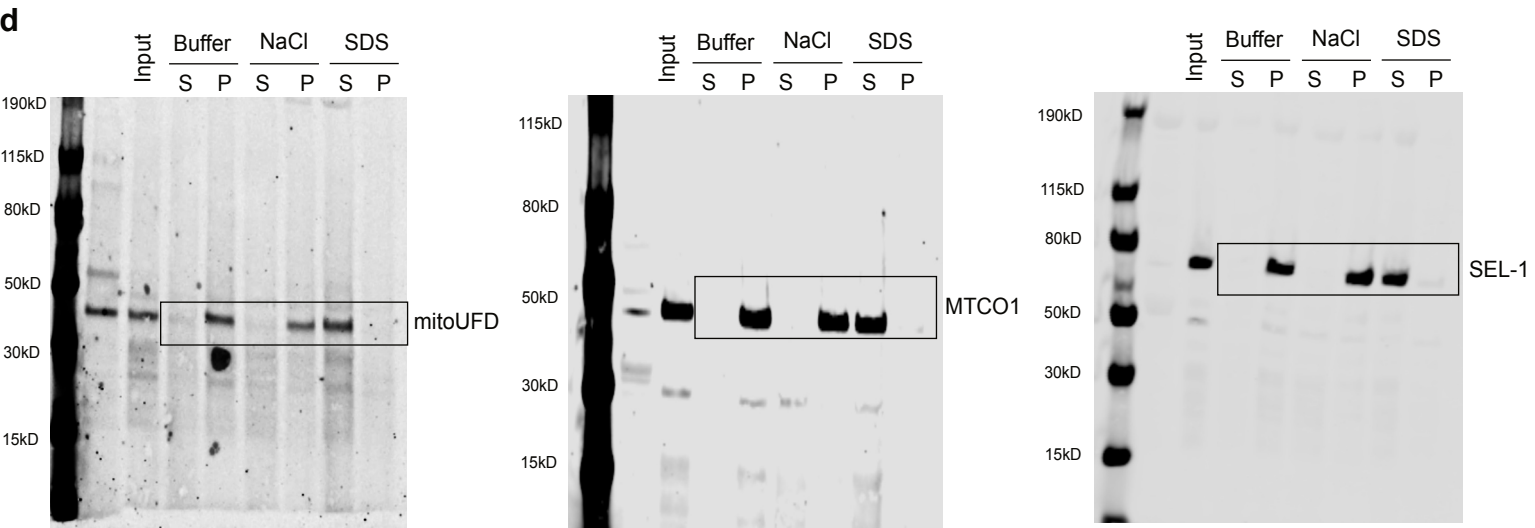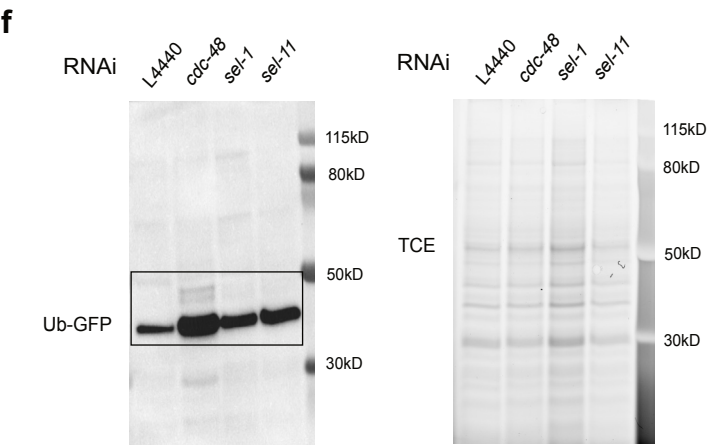

Supplement: Supplementary file 20 — Uncropped blots. [file 41556_2025_1799_MOESM20_ESM.pdf]

uncropped blots related for Extended Data Fig. 8

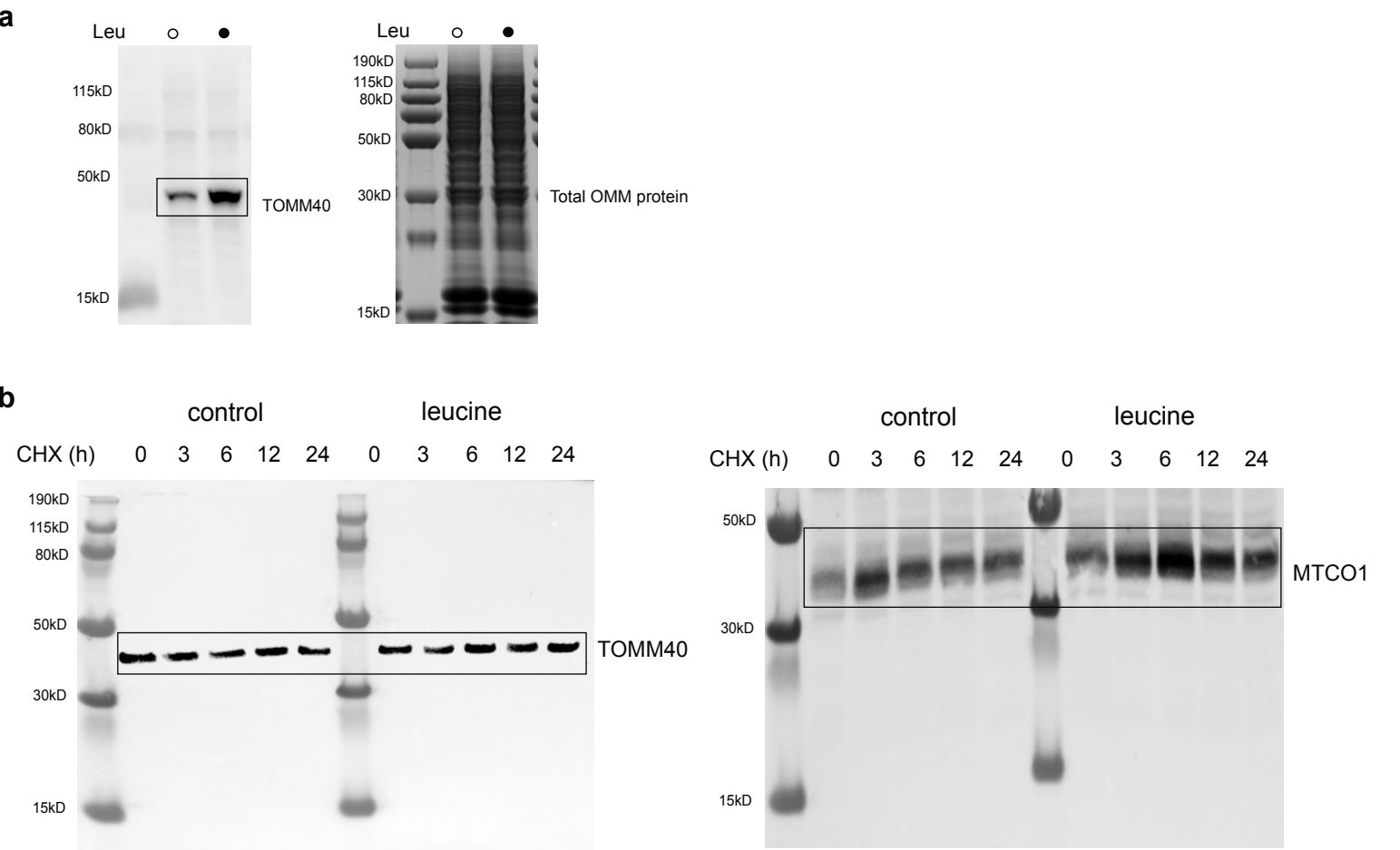

Supplement: Supplementary file 23 — Uncropped blots. [file 41556_2025_1799_MOESM23_ESM.pdf]

**c**

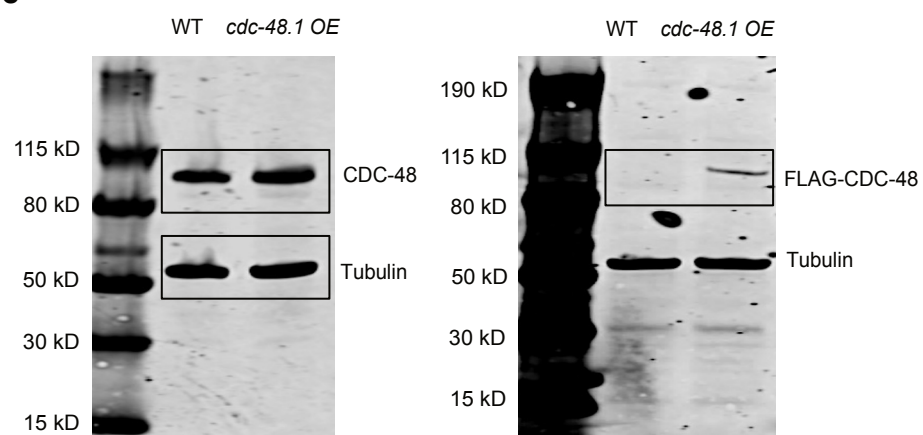

Supplement: Supplementary file 25 — Uncropped blots. [file 41556_2025_1799_MOESM25_ESM.pdf]
